# The relationship of E-selectin single-nucleotide polymorphisms with breast cancer in Iraqi Arab women

**DOI:** 10.5808/gi.22042

**Published:** 2022-12-13

**Authors:** Bilal Fadil Zakariya, Asmaa M. Salih Almohaidi, Seçil Akıllı Şimşek, Safaa A. Al-Waysi, Wijdan H. Al-Dabbagh, Areege Mustafa Kamal

**Affiliations:** 1 Department of Biology, Institute of Sciences, Çankiri Karatekin University, Çankırı Merkez 18100, Turkey; 2 Department of Biology, College of Science for Women, University of Baghdad, Baghdad 10022, Iraq; 3 Department of Microbiology, Medical City Teaching Hospital, Baghdad 10011, Iraq; 4 Department of Oncology, Medical City Teaching Hospital, Baghdad 10011, Iraq; 5 Department of Pathology/Oncology, Medical City Teaching Hospital, Baghdad 10011, Iraq

**Keywords:** breast neoplasms, E-selectin, expression, polymorphism

## Abstract

Breast cancer (BC) is a significant threat to female health, with both modifiable and non-modifiable risk factors. It is essential to monitor patients regularly and to raise population awareness. Increasing research also suggests that E-selectin (SELE) may increase tumor angiogenesis and the development of cancer. This study investigated *SELE* single-nucleotide polymorphisms (SNPs) in the following positions: rs5367T/C, rs5368C/T, rs5362T/G, and rs5362T/C. Using polymerase chain reaction, significant differences in allele and genotype frequencies were found between BC patients and controls. Position rs5368 was associated with an increased risk of BC for the CT and TT genotypes, with odds ratios (ORs) of 16.3 and 6.90 (Fisher probability = 0.0001, p = 0.005). Women with the T allele had a 19.3-fold higher incidence of BC, while allele C may be a protective allele against BC (OR, 0.05). Heterozygous genotypes at rs5367, rs5362, and rs5362 were significantly more common in BC patients, with ORs of 5.70, 4.50, and 3.80, respectively. These SNPs may be associated with the risk of BC, because the frequency of mutant alleles was significantly higher in patients (OR: 4.26, 3.83, and 4.30, respectively) than in controls (OR: 0.23, 0.30, and 0.20, respectively). These SNPs may be considered a common genotype in the Iraqi population, with the wild-type allele having a protective fraction and the mutant allele having an environmental fraction. The results also revealed a 2-fold increase in gene expression in BC patients compared to controls, with a significant effect (p = 0.017). This study's findings confirm the importance of *SELE* polymorphisms in cancer risk prediction.

## Introduction

Breast cancer (BC) is becoming a significant threat to female health in Iraq, where it is the second leading cause of death after cardiovascular diseases among Iraqi women. In 2018, there were 6,094 new cases of BC, accounting for 34.06% of all female cancers; the highest incidence of BC was seen in middle-aged women (45–49 years old), while the peak age-specific incidence was documented in women 50-54 years old according to the Iraqi Cancer Registry of the Ministry of Health [1].

Polymorphisms related to the gene encoding E-selectin (SELE) are interesting for two reasons: firstly, they are overrepresented in some individuals with a disease related to increased leukocyte adhesion in the endothelium, and secondly, these are functional polymorphisms that change ligand attraction [2]. Polymorphisms alter the specificity of *SELE* binding, resulting in increased function under influx conditions and possibly increasing the number of white blood cells rolling and adhering to the endothelium [3]. Some case-control studies have found that inherited genetic factors play a substantial role in cancer risk [4]. Several single-nucleotide polymorphisms (SNPs) have been discovered inside the *SELE* gene; for instance, women carrying the *SELE* S128R polymorphism, which results in an amino acid change (from serine to arginine), had a significantly increased risk of BC in an Asian population. The effects of the S128R polymorphism on BC susceptibility may be population-dependent [5]. This suggests that it is a functional polymorphism. Furthermore, previous research has shown that *SELE* polymorphisms play a role in susceptibility to carcinoma development to some extent [6].

Endothelial cells express *SELE*, a key adhesion molecule known for its involvement in facilitating cell-cell contacts between cancer cells and endothelial monolayers during metastasis [7]. The adherence of circulating malignancies to the vascular endothelium is crucial during the early stages of metastasis; *SELE* plays a critical role in adhesion with extravasation of leukocytes conveying ligands (s-Lex) or (s-Lea) in damaged areas of tissues. Existing information has led to an increased interest in research exploring new diagnostic factors. Therefore, this study aimed to investigate the possible links between genetic variants in the *SELE* gene and BC risk in the Iraqi population.

## Methods

The study was approved by the Medical City-Iraq directorate's ethical committee, and 100 women who visited the oncology teaching hospital's breast clinic were recruited. Group 1 consisted of 60 women recently diagnosed with BC who provided informed written consent. The diagnosis of BC in the patients was based on histopathological results obtained from hospital files and tissue block processing. Group 2 consisted of 40 healthy women who served as controls. Patients with other systemic diseases and those taking hormone-modifying medications were excluded from the study. Information was collected on age (years), family history, body mass index (kg/m^2^), menopausal status, smoking tobacco, breastfeeding, menstrual cycle regularity, and other factors relevant for BC.

### DNA/RNA extraction

Each patient and healthy control had 8–10 mL of blood drawn. First, blood samples were obtained from the cubital vein and then divided into two aliquots: one for the gene expression, for which TRIzol was used to preserve the blood samples [8], and the other aliquot was directly into an EDTA-containing tube for genotyping the selected SNPs. DNA/RNA extraction was then performed using a Norgen Biotek kit (Thorwold, ON, Canada) according to the manufacturer's guidelines. The quantity and quality of extracted DNA/RNA were investigated based on the ratio of optical density at the 260 and 280 nm wavelengths using (Qubit 4, Invitrogen, Waltham, MA, USA).

### Polymorphism analysis

A total reaction volume of 25 μL, containing 3 μL of DNA sample, 7.5 μL of nuclease-free H2O, 12.5 μL of polymerase chain reaction (PCR) master mix containing MgCl_2_, a dNTP mix (Promega, Madison, WI, USA) and 2 μL of primer. Two primers were used in this study (Table 1), and as a result, two PCR tests performed on each DNA sample were used for genotyping the SNPs. The PCR reaction was performed using a thermocycler (Applied Biosystems, Waltham, MA, USA) s as follows: the sample was initially denaturation at 95°C for 5 min, 45 cycles of denaturation at 94°C for 30 s, annealing at 57°C for 30 s; shadowed extension at 72°C for 40 s, final extension at 72°C for 10 min, and holding at 8°C. Amplification was screened using a Sanger sequencing panel (Macrogen, Seoul, Korea) to detect variation.

### Analysis of *SELE* gene expression

#### Reverse transcription PCR assay

Total RNA was transcribed using a High-Capacity cDNA Reverse Transcription Kit (Thermo Fisher, Waltham, MA, USA) with an RNase inhibitor, according to the manufacturer's instructions. PCR reactions were carried out in a 20 μL volume, containing 10 μL of 2× Go-Taq Green master mix (Promega), 3 μL of cDNA, 5 μL of nuclease-free water, 1 μL of forward primer, and 1 μL of the reverse primer (Table 2). The reaction was carried out using a thermocycler (Bioneer, Daejeon, Korea). The quantitative PCR conditions were as follows: initial denaturation at 95°C for 5 min, followed by 30 cycles of denaturation at 95°C for 30 s and annealing/extension at 55°C for 60 s, followed by a melting curve at 1°C-intervals from 55°C to 95°C.

### Statistical analysis

*SELE* genotypes and gene expression were statistically analyzed using SPSS version 25 (IBM Corp., Armonk, NY, USA). Allele frequencies of the *SELE* gene were calculated by the direct gene counting method via Geneious Software Prime 11.1.5 (Auckland, New Zealand). At the same time, the significance of the departure from Hardy-Weinberg (H-W) equilibrium was estimated using the H-W calculator for two alleles, which is available free online [9]. The Pearson chi-square test was used to assess the significance of differences between the observed and expected frequencies. Alleles and genotypes of *SELE* were presented as percentages and frequencies, and the two-tailed Fisher exact probability test was used to evaluate the significance of differences between their distributions in BC patients and controls. In addition, odds ratios (ORs) were also estimated to define the association between *SELE* alleles and genotypes with the disease; an OR less than 1 indicates a negative association, while an OR more than 1 indicates a positive association. Differences in clinical characteristics between BC patients and healthy controls were examined using the independent-sample t-test. A value of p = 0.05 was deemed to indicate statistical significance.

## Results

### Clinical characteristics

Table 3 shows the characteristics of the patients and controls. The statistical analysis revealed significant differences between the patient and control groups in terms of age (p = 0.001), body mass index (BMI) (p < 0.001), antiperspirant use (p = 0.014), breastfeeding (p = 0.005), family history (p < 0.001), smoking (p = 0.045), menopause age (p = 0.034), and menstrual cycle regularity (p < 0.001). We did not find significant differences between patient and control groups regarding radiation and contraceptive pills.

**Fig. 1.**
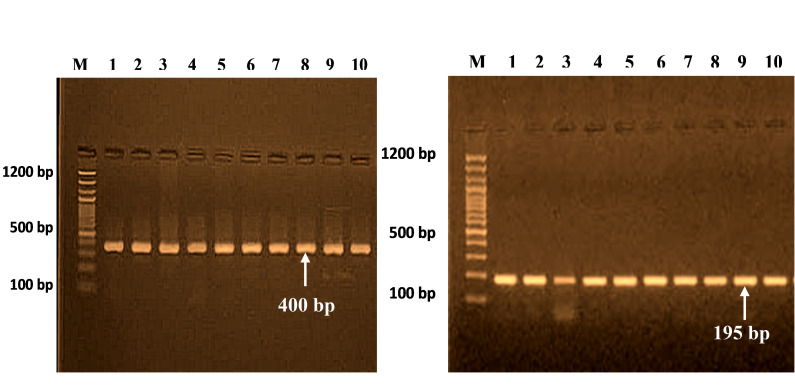
PCR products for *SELE* gene (400 bp) and (195 bp) on 2% agarose gel at 70 V for 2 h. M, 100 bp DNA ladder.

The PCR cycles for 400-bp and 195-bp segments are shown in Fig. 1, corresponding to four polymorphisms at different positions of the *SELE* gene (Table 4).

### Polymorphism distribution and frequency of alleles in *SELE* at rs5367 (T>C)

**Fig. 2.**
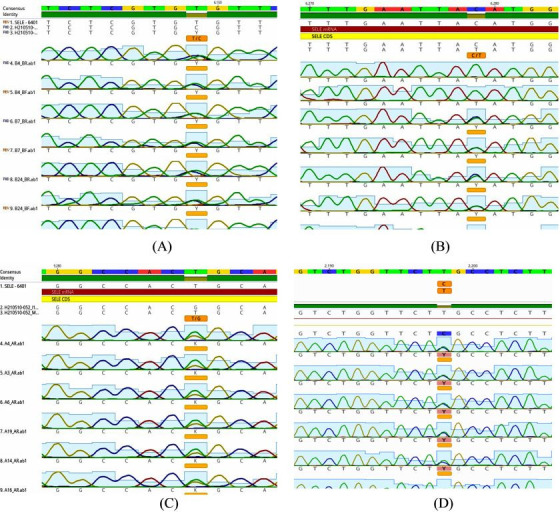
(A-D) Alignment sequence of the *SELE* gene in breast cancer patients and healthy control with reference sequencing in National Center for Biotechnology Information NCBI by Geneious Software Prime 11.1.5 (Auckland, New Zealand).

This study analyzed the distribution of the genotype and allele frequencies of the rs5367 polymorphism in patients and controls (Table 5). This polymorphism presents three genotypes (TT, TC, and CC) corresponding to two alleles (T and C) (Fig. 2A). The genotype frequencies in both groups agreed with H-W equilibrium, and there were no significant differences between the observed and expected frequencies in both patients and controls (χ^2^ = 2.7 and χ^2^ = 0.2, respectively).

The TT wild-type homozygous genotype frequency was significantly higher in controls than in patients (86.7% vs. 53.3%), reflecting a negative association with BC (OR, 0.18). The CC mutant genotype was not present in any patients or controls (0%). However, the TC heterozygous genotype frequency was significantly higher in patients (46.7%) than in controls (13.3%) (OR, 5.70; Fisher probability, 0.001; p = 0.007). The T allele frequency was less common in patients than in controls (76.7% vs. 93.3%), while the C allele frequency was higher (23.3% vs. 6.70%). A negative association was found for the T allele (OR, 0.18) and a positive association for the C allele (OR, 4.25) (for both: Fisher exact probability=0.02; p = 0.016) (Table 6).

### Polymorphism distribution and frequency of alleles in *SELE* at rs5368 (C>T)

This study also investigated the genotype and allele frequency distributions of the rs5368 polymorphism in patients and controls (Table 5). This polymorphism in BC patients and control produced three genotypes (CC, CT, and TT), which corresponded to two alleles (C and T) (Fig. 2B). The genotype frequencies in both groups agreed with H-W equilibrium, and there were no significant differences in observed and expected frequencies in either patients or controls (χ^2^ = 0.4 and χ^2^ = 0.03, respectively).

The frequency of the CC wild-type homozygous genotype in controls (90.0%) was also significantly higher than in patients (33.3%), with a negative association (OR, 0.03). The TT mutant genotype was significantly less common in patients than in controls (13.3% vs. 0%), with a positive association (OR, 6.90). However, the CT heterozygous genotype frequency was significantly higher in patients (53.3%) than in controls (10.0%) (OR, 5.70; Fisher probability, 0.01; p = 0.005). The C allele frequency was lower in patients than in controls (60.0% vs. 96.7%), while the T allele frequency was higher (40.0% vs. 3.30%). The C allele had a negative association (OR, 0.05), and the T allele had a positive association (OR, 19.3). Both variations were highly significant (Fisher probability, 0.0001; p < 0.001) (Table 6).

### Polymorphism distribution and frequency of alleles in *SELE* at rs5361 (T>G)

The genetic polymorphism of *SELE* was investigated at position rs5361, which presented with three genotypes (TT, TG, and GG) that corresponded to two alleles (T and G) (Fig. 2C). The results for patients and controls were in agreement with the expected H-W equilibrium, and there was no significant difference between the observed and expected frequencies in patients (χ^2^ = 1.2) and controls (χ^2^ = 0.08) (Table 5).

At the position rs5361 in *SELE*, patients had a significantly higher frequency of the TG genotype than controls (33.3% vs. 9.0%; OR, 4.50; Fisher's probability, 0.05; p = 0.03). In contrast, the frequency of the TT wild-type genotype was significantly lower in patients than in controls (66.7% vs. 91.0%; OR, 0.22). The G allele frequency was significantly higher in patients than in controls (16.7% vs. 5.0%; Fisher probability, 0.04). A positive association was found for the G allele (OR, 3.83) and a negative association for the T allele (OR, 0.30), which was a significant difference (p = 0.052) (Table 6).

### Polymorphism distribution and frequency of alleles in *SELE* at rs5362 (T>C)

Additionally, the genotype and allele frequency distributions of the rs5362 polymorphism in both patients and controls were investigated (Table 5). This polymorphism in BC patients and control produced three genotypes (TT, CT, and CC), which corresponded to two alleles (T and C) (Fig. 2D). The genotype frequencies in both groups were consistent with H-W equilibrium, and neither patients nor controls showed appreciable differences between the observed and anticipated frequencies (χ^2^ = 0.13 and χ^2^ = 0.24, respectively).

However, at position rs5362, patients had a significantly higher frequency of the TC genotype than controls (43.3% vs. 16.7%), with a positive association (OR, 3.80; Fisher probability, 0.04; p = 0.029). In contrast, the frequency of the TT wild-type genotype was significantly lower in patients than in controls (50.0% vs. 83.3%; OR, 0.20). The frequency of the CC mutant homozygous genotype in patients (6.70%) was also significantly higher than in controls (0%), with a positive association (OR, 5.40). Therefore, the C allele frequency was significantly higher in patients than in controls (28.3% vs. 8.30%) (Fisher's probability, 0.008). A positive association was found for the C allele (OR, 4.30) and a negative association for the T allele (OR, 0.20), corresponding to a highly significant difference (p = 0.007) (Table 6).

### Quantification of *SELE* gene expression

**Fig. 3.**
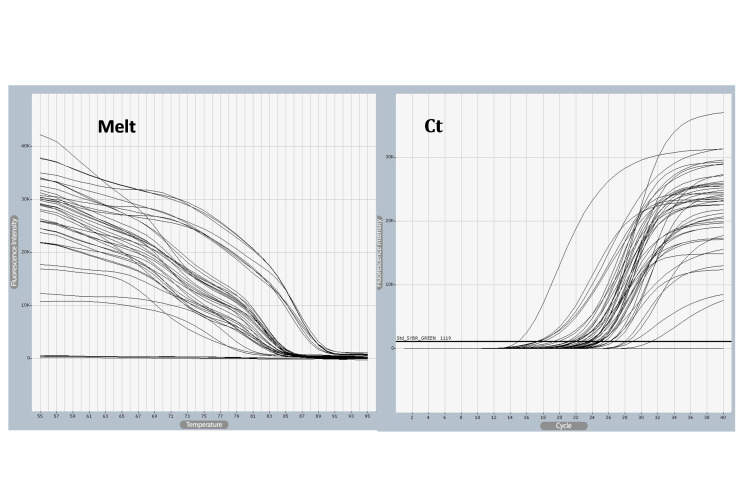
*SELE* gene amplified using quantitative polymerase chain reaction (qPCR) samples. Ct values varied from 18 to 31and melting temperature varied from 25°C to 55°C, The photograph was obtained directly from the qPCR machine.

**Fig. 4.**
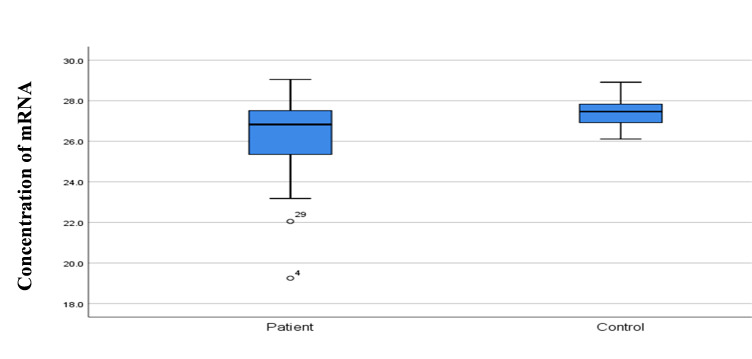
Comparison of concentration RNA among control and study groups.

*SELE* expression was higher in BC patients than in controls, with a statistically significant 2-fold increase in gene expression value (2.06 vs. 1.01). The patient group had a higher copy number of mRNAs (Figs. 3 and 4). The Ct values in the patient group varied from 19.33 to 26.27, with a mean of 26.27, while those of the controls ranged from 26.13 to 28.02, with a mean of 27.43. As shown in Table 7, there was a significant difference between these groups in terms of the mean Ct value of *SELE* (p = 0.017).

## Discussion

In the current case-control study, we identified several risk factors for BC in a population of Iraqi women. Age, BMI, genetic predisposition, smoking, and the menstrual cycle are all relevant risk factors for BC [10]. A link was found between a BMI of 25 kg/m^2^ and an increased risk of BC [11]. These mechanisms include androgen to estrogen conversion in adipose tissue [12], as well as inflammation and metabolic risk factors for cancer [13] and epigenetic modifications to genes such as *BRCA1* [14]. A family history of BC has been linked to a 4- to 5-fold increased risk of the disease, implying a strong genetic component and possibly stronger associations with specific genes. An epidemiological study [10] found that having a history in first- and second-degree relatives increased the risk by 80%. BC has been found to be strongly related to menopause age [15]. The hormonal signaling that occurs during the ovaries' active period remains the most compelling explanation for the latter finding [16]. Breastfeeding's role in BC prevention has also been discussed [17].

The present study investigated SNPs of *SELE* at rs5367, rs5368, rs5362, and rs5362, which are the most frequently, studied positions. The patients and control group showed agreement with H-W equilibrium in all genotypes. This agreement may occur because these SNPs may be considered common genotypes in the Iraqi population, with wild-type alleles having protective fraction susceptibility and the mutant allele having an environmental fraction. This result aligns with a previous study on type 2 diabetes mellitus [18]. Other Iraqi studies have emphasized that heterozygous genotypes in different SNPs of the *SELE* gene could be a risky genotype for type 2 diabetes mellitus in the Iraqi population [19]. However, these SNPs could also confer an elevated risk for other diseases such as BC.

The present study found that the genotypes and alleles of *SELE* significantly varied between BC patients and controls. The rs5367 TC heterozygous genotype was most common among BC patients. This genotype was present in about 46.7% of BC cases, but in only 13.3% of controls, with significance according to Fisher probability (0.01) and a positive association with the disease (OR, 5.70). This observation may highlight the role of the *SELE* polymorphism in disease pathogenesis because present results showed that the wild-type allele T had a protective effect against BC (OR, 0.23), indicating a negative association with BC. In contrast, the mutant allele C had an etiological impact (OR, 4.26), with a positive association with disease; therefore, females who carry allele C of rs5367 may be more susceptible to BC than females who have allele T. The rs5368 CT and TT genotypes recorded the highest frequencies in BC patients, accounting for about 66% of BC cases. This observation may highlight the role of the *SELE* rs5368 polymorphism in disease pathogenesis because the wild-type genotype (CC) was observed to have a low frequency in patients (about 33%). The T allele was identified as an environmental effect allele with a positive association with BC (OR, 19.3). In contrast, the C allele had a preventive fraction, with a negative association with BC (OR, 0.05), because the CC genotype showed the highest frequency in the control group (90%). High significance was also found for the CT and TT genotypes at position rs5368, suggesting that this polymorphism plays a role in cancer development.

The TG genotype frequency at position rs5361 was also higher in patients than in controls, whereas the GG genotype showed a lower frequency in both the control group and patients (0%). This SNP may be considered a common genotype in the Iraqi population, with the wild-type allele T having a protective fraction (OR, 0.30) and the mutant allele G having an environmental fraction (OR, 3.83). SNP rs5362 showed significant variation between BC patients and controls. The TC and CC genotypes recorded a higher frequency in BC patients (50%) than in controls (16%), with a positive association with disease (OR, 3.80 and 5.40), respectively. This observation may suggest the role of the rs5362 polymorphism in disease because the TT wild-type genotype was recorded at a high frequency in the control group (83.3%). The results of this study suggest that mutant C allele may have an environmental impact on the Iraqi population, with a positive correlation with BC (OR, 4.30), whereas the wild-type allele T exhibited a negative correlation with BC (OR, 0.20).

The *SELE* polymorphism results revealed no significant differences in allele or genotype frequencies between BC patients and controls, but it was interesting to note that the heterozygous genotypes at rs5367, rs5368, rs5361, and rs5362 were significantly increased in BC patients, and all heterozygous genotypes accounted for 46.7%, 53.3%, 33.3%, and 43.3%, respectively of BC cases, with positive associations shown by ORs of 5.70, 16.3, 4.50, and 3.80, respectively. These findings show that these polymorphisms may be linked to the development of BC, because the mutant alleles may have an environmental effect in Iraqi women patients, in contrast to wild-type genotypes, which showed a very high frequency in the patient and control groups, with the wild-type alleles likely having a preventive fraction against disease.

*SELE* genetic variations have been shown to play a crucial role in increasing the risk of various diseases. According to Kontogianni et al. (2013) [20], the *SELE* rs5361 SNP may enhance the chance of developing pancreatic and stomach malignancies. The *SELE* S128R polymorphism, according to Alessandro et al. [2], might change tumor-endothelial interactions and neoplastic cell motility, which may modulate the metastatic phenotype [21]. Furthermore, in Malaysians, the *SELE* S128R gene polymorphism has been related to BC, as well as linked to a higher risk of relapse and death in colorectal cancer patients [5]. *SELE* polymorphisms (A561C) and (G98T) were also found to be strongly related to an elevated risk of coronary heart disease [22]. According to Xia et al. (2012) [23], SNPs in immunoregulatory genes may influence the risk of gastric cancer. Functional polymorphisms such as rs5353 may also play a role in cystic fibrosis penetrants [24]. Furthermore, the current study discovered that *SELE* gene expression was significantly elevated in the blood of BC patients compared to controls, with a 2-fold increase in gene expression (2.06 vs. 1.01) compared to controls (1.01). Our findings show that *SELE* is likely associated with BC development because an avascular adhesion cascade occurs around the malignant transformation region, which is associated with *SELE* expression and increased activity, assisting in snip-resistant adhesion and the transendothelial movement of circulating carcinoma target tissues [25].

According to O'Hanlon et al. (2002) [26], *SELE* is involved in BC cell adhesion and plays a significant role in cancer cell dispersion. It also influences carcinoma cell aggregation and interactions with endothelial cells [26]. *SELE* levels are raised in ovarian carcinoma [27], leukemia [28], and lung carcinoma [29]. Furthermore, the present result from Iraqi patients aligns with previous studies about Iraqi patients with other diseases (e.g., type 2 diabetes mellitus) that showed a positive association with *SELE* polymorphisms, and these local studies all concluded that *SELE* could be a risk factor [11]. The observations of the present study suggest that evaluating the adhesion molecule *SELE* in females with BC may be added to the panel of assays used as a factor to predict patients’ prognosis and monitor disease progression.

## Figures and Tables

**Table 1.** Primers for the *SELE* gene used and designed in this study

Name of primers	Sequence of primers	Product size (bp)	Design by
Primer 1			
*SELE* forward	GCGCTACTTAGTTTTCAGCATGT	400	Second author
*SELE* reverse	ACTTGGTTACCTTGGGAAACG		
Primer 2			
*SELE* forward	AGTAATAGTCCTCCTCATCATG	195	First author
*SELE* reverse	ACCATCTCAAGTGAAGAAAGAG		

**Table 2.** Primers for *SELE* gene expression

Name of primers	Sequence of primers	Product size (bp)	Design by
Primers for the *SELE* gene			
*SELE* forward	CCTGCAATGTGGTTGAGTGTG	156	Second author
*SELE* reverse	CTCGTTGTCCCAATTCCCAGA		
Primers for the housekeeping gene			
B-actin forward	ATGCTTCTAGACGGACTGCG	110	First author
B-actin reverse	GTTTCAGGAGGCTGGCATGA		

**Table 3.** The observed frequencies and percentage of risk factors related to the clinical characteristics of the patient and control groups

Characteristic	No. (%)		Test result p-value
	Case	Control	
Age (yr)			
≤35	45 (75.0)	16 (40.0)	0.001**
>35	15 (25.0)	24 (60.0)	
Body mass index			
Underweight	0	1 (2.50)	<0.001***
Normal	13 (21.7)	19 (47.5)	
Overweight	17 (28.3)	13 (32.5)	
Obese	29 (48.3)	7 (17.5)	
Antiperspirant use			
User	23 (38.3)	6 (15.0)	0.014*
Non-user	37 (61.7)	34 (85.0)	
Breastfeeding			
Yes	27 (45.0)	7 (17.5)	0.005**
No	33 (55.0)	33 (82.5)	
Family history of breast cancer			
Found	38 (63.3)	3 (12.5)	<0.001***
First degree	17 (44.7)	3 (12.5)	
Second degree	21 (55.3)	2 (92.5)	
Not found	22 (36.7)	37 (92.5)	
Smoking			
Smoker	11 (18.3)	0	0.044*
Non-smoker	49 (81.7)	40 (100)	
Menopause age			
Yes	15 (25.0)	3 (12.5)	0.034*
No	45 (75.0)	37 (92.5)	
Menstrual cycle			
Irregular	33 (55.0)	1 (2.5)	<0.001***
Regular	27 (45.0)	39 (97.5)	
Radiation			
Exposed	4 (6.60)	0	0.215 NS
Non-exposed	56 (93.3)	40 (100)	
Contraceptive pills			
User	15 (25.0)	9 (22.5)	0.804 NS
Non-user	45 (75.0)	31 (77.5)	

Significant *p < 0.05, **p < 0.01, ***p < 0.001; NS, non-significant.

**Table 4.** Position and allele information of *SELE* polymorphisms in NCBI assembly data

SNP	RefSeqGene	Gene (ID)	SNP to RefSeqGene	Effect
rs5367 T>C	NG_012124.1	SELE (6401)	Fwd	N/A
rs5368 C>T	NG_012124.1	SELE (6401)	Fwd	H (His) > Y (Tyr)
rs5361 T>G	NG_012124.1	SELE (6401)	Rev	S (Ser) > C (Cys)
rs5362 T>C	NG_012124.1	SELE (6401)	Fwd	N/A

SNP, single nucleotide polymorphism; Fwd, forward; Rev, reverse; N/A, not applicable.

**Table 5.** Hardy-Weinberg equilibrium expected versus observed *SELE* genotype frequencies

	Patients		Control	
	Observed	Expected	Observed	Expected
Distribution of SNP rs5367				
TT	16	17.6	26	26.1
TC	14	10.7	4	3.7
CC	0	1.6	0	0.1
*T*	0.77	ND	0.93	ND
*C*	0.23	ND	0.07	ND
*X^2^*	2.7 NS	-	0.2 NS	-
Distribution of SNP rs5368				
CC	10	10.8	28	28
CT	16	14.4	2	1.9
TT	4	4.8	0	0
*C*	0.6	ND	0.97	ND
*T*	0.4	ND	0.03	ND
X^2^	0.4 NS	-	0.03 NS	-
Distribution of SNP rs5361				
TT	20	20.8	27	27.1
TG	10	8.3	3	2.9
GG	0	0.8	0	0.1
*T*	0.83	ND	0.95	ND
*G*	0.17	ND	0.05	ND
X^2^	1.2 NS	-	0.08 NS	-
Distribution of SNP rs5362				
TT	15	15.4	25	25.2
TC	13	12.2	5	4.6
CC	2	2.4	0	0.2
*T*	0.72	ND	0.92	ND
*C*	0.28	ND	0.08	ND
X^2^	0.13 NS	-	0.24 NS	-

SNP, single nucleotide polymorphism; ND, not detected; NS, not significant.

**Table 6.** Distribution of genotype and allele frequencies for several *SELE* single-nucleotide polymorphisms in breast cancer patients and controls

Genotype	No. (%)		OR (95% CI)	Fisher exact probability*	p-value
	Patients (G1)	Controls (G2)			
*SELE* rs5367 genotype					
T/T	16 (53.3)	26 (86.7)	0.18 (0.05–0.62)	0.01	0.008**
T/C	14 (46.7)	4 (13.3)	5.70 (1.59–20.33)	0.01	0.007**
C/C	0	0	-	NS	NS
Allele					
T	46 (76.7)	56 (93.3)	0.23 (0.07–0.76)	0.02	0.016*
C	14 (23.3)	4 (6.7)	4.26 (1.3–13.8)	0.02	
*SELE* rs5368 genotype					
C/C	10 (33.3)	28 (90.0)	0.03 (0.01–0.18)	0.0001	<0.001***
C/T	16 (53.3)	2 (10.0)	16.3 (3.2–77.59)	0.0001	0.005**
T/T	4 (13.3)	0	6.90 (0.35–136.8)	0.05	0.122 ^NS^
Allele					
C	36 (60.0)	58 (96.7)	0.05 (0.01–0.23)	0.0001	<0.001***
T	24 (40.0)	2 (3.30)	19.3 (4.3–86.8)	0	
*SELE* rs5361 genotype					
T/T	20 (66.7)	27 (91.0)	0.22 (0.05–0.91)	0.05	0.037*
T/G	10 (33.3)	3 (9.0)	4.50 (1.1–18.50)	0.05	0.040*
G/G	0	0	-	NS	NS
Allele					
T	50 (83.3)	57 (95.0)	0.30 (0.07–1.01)	0.07^NS^	0.052*
G	10 (16.7)	3 (5.0)	3.83 (1.0–14.58)	0.04	
*SELE* rs5362 genotype					
T/T	15 (50.0)	25 (83.3)	0.20 (0.06–0.66)	0.007	0.008**
T/C	13 (43.3)	5 (16.7)	3.80 (1.2–12.7)	0.04	0.029*
C/C	2 (6.7)	0	5.40 (0.24–116.3)	0.4^NS^	0.285 NS
Allele					
T	43 (71.7)	55 (91.7)	0.20 (0.08–0.67)	0.008	0.007**
C	17 (28.3)	5 (8.3)	4.30 (1.5–12.72)	0.008	

OR, odds ratio; CI, confidence interval; NS, non-significant. Significant *p < 0.05, **p < 0.01, ***p < 0.001.

**Table 7.** Evaluation of *SELE* gene expression among study groups

Group	Mean±SD of Ct value	Range	ΔCt	Fold change of gene expression
Patient group (n = 60)	26.27 ± 0.39	19.3–29.1	–1.01	2.06
Control group (n = 40)	27.43 ± 0.30	26.1–28.02	0.36	1.01
p-value	0.017		-	-

SD, standard deviation.
